# Floating Drug Delivery of Nevirapine as a Gastroretentive System

**DOI:** 10.4103/0975-1483.71622

**Published:** 2010

**Authors:** Hari BN Vedha, Reddy A Brahma, Rani B Samyuktha

**Affiliations:** *Department of Pharmaceutics, CARISM, SASTRA University, Thirumalaisamudram, Thanjavur - 613 401, Tamil Nadu, India*

**Keywords:** Floating beads, gastroretentive, nevirapine

## Abstract

A multiple-unit floating drug delivery system based on gas formation technique was developed, in order to prolong the gastric residence time and to increase the overall bioavailability of the dosage form. The floating bead formulations were prepared by dispersing nevirapine together with calcium carbonate in a mixture of sodium alginate and hydroxypropyl methylcellulose solution and then dripping the dispersion into an acidified solution of calcium chloride. Calcium alginate beads were formed, as the alginate underwent ionotropic gelation by calcium ions, and carbon dioxide developed from the reaction of carbonate salts with acid. The obtained beads were able to float due to CO_2_-gas formation and the gas entrapment by the polymeric membrane. The prepared beads were evaluated for percent drug loading, drug entrapment efficiency, morphology, surface topography, buoyancy, *in-vitro* release, and release kinetics. The formulations were optimized for different weight ratios of the gas-forming agent and sodium alginate. The beads containing higher amounts of calcium carbonate demonstrated an instantaneous, complete, and excellent floating ability over a period of 24 hours. The increased amount of the gas forming agent did not affect the time to float, but increased the drug release from the floating beads, while increasing the coating level of the gas-entrapped membrane, increased the time to float, and slightly retarded the drug release. Good floating properties and sustained drug release were achieved. Finally, these floating beads seemed to be a promising gastroretentive drug delivery system.

## INTRODUCTION

Drug absorption from a gastrointestinal tract (GI) is a complex procedure and is subjected to many variables.[1] These variables make the *in-vitro* performance of the drug delivery systems uncertain.[2] The process and ability to prolong and control the emptying time is a valuable asset for dosage forms, which reside in the stomach for a longer period of time than the conventional dosage forms,[3] such as, tablets, capsules, and granules. These physiological problems have been overcome by several drug delivery systems, by investigating the prolonged gastric retention time.[4,5] Attempts are being made to develop a controlled drug delivery system that can provide therapeutically effective plasma drug concentration levels for longer durations, thereby reducing the dosing frequency and minimizing fluctuations in the plasma drug concentration at a steady state by delivering the drug in a controlled and reproducible manner.[6]

On the basis of the mechanism of mucoadhesion,[7,8] flotation,[9] sedimentation,[10,11] expansion,[12] modified shape system[13,14] or by the simultaneous administration of pharmacological agents,[15,16] the controlled gastric retention of solid dosage forms may be achieved, which delay gastric emptying. In addition to this, a wide variety of both natural and synthetic hydrophilic polyionic systems such as alginates have been investigated for the preparation of multiple unit floating dosage forms (FDF’s).[6]

In the present study, a multiple-unit FDF was designed keeping in view the ‘all or nothing’ response of single-unit systems.[17] Literature review indicates a widespread use of sodium alginate for achieving the sustained release of drugs,[18,19] as it targets the gastric mucosa[20,21] and increase the bioavailability of the drugs[22] because of its ability to form a stable and bioadhesive gel with calcium ions.[23] Hydroxy propyl methyl cellulose (HPMC) has been reported to enhance the sustained release properties of alginate by providing a denser inner matrix.[24] Also the preparative methodology of alginate beads involves the use of aqueous solvents, avoiding exposure of the ingredients to high temperatures and toxic organic solvents;[25–27] moreover, the resulting preparation is non-immunogenic, with bioadhesive properties that could serve as a potential advantage in stomach targeting.

The efficacy and safety of non-nucleoside reverse transcription inhibitor (NNRTI)-based regimens have been demonstrated in the treatment of HIV-I infected patients. The World Health Organization (WHO) recommends the use of an NNRTI-based regimen as a first line-anti-retroviral treatment in source-limited settings, specifically; experiences with a generic fixed-dose combination (FDC) with nevirapine (NV) have been widely documented in resources-limited settings. The mechanism of action is that it binds directly to the human immunodeficiency virus type I (HIV-I) RT, an RNA-dependent DNA polymerase, blocking its function in viral DNA replication.

## MATERIALS AND METHODS

Nevirapine (NV) was obtained as a gift sample from (Hetero Drugs Pvt. Ltd., Jidimetla, Hyderabad, India), Sodium alginate (Loba Chemie Pvt. Ltd., Mumbai, India), Hydroxy propyl methyl cellulose (Hymedia Laboratories Pvt. Ltd., Mumbai, India), Calcium chloride (Fischer Chemie Ltd., Chennai, India), Calcium carbonate (Paxmy Speciality Chemicals, Chennai, India), all other reagents and chemicals were of analytical grade.

### Method of preparation of floating beads of nevirapine

According to the published procedure,[28] the modified procedure for preparing floating beads was as follows; 0.2 gm of nevirapine was dissolved in 15 ml of methanol. This solution was dispersed in 12.5 ml (6 Wt/ Vol) of alginate solution containing HPMC (9:1) and then the gas forming agent CaCO_3_ was added to the solution in weight ratios of 0 : 1 - 1 : 1 (CaCO_3_: Alginate w/w) [Table 1]. Then the resulting solution was dropped through an 18 h (0.8 × 30 mm) syringe needle into 50 ml of CaCl_2_ solution, (15% w/v), along with 10% v/v acetic acid. The beads were allowed to remain in the same solution for two hours to improve their mechanical strength. Next the beads were washed, initially with ethanol and subsequently with distilled water, and then freeze dried.

**Table 1:** Formulation variables and evaluation parameters of various Nevirapine floating bead formulations (NV-No gas forming agent, NV1-0.5% gas forming agent, NV2-0.75% gas forming agent, NV3-1:1 gas forming agent)

Formulation	Calcium carbonate: Sodium Alginate (wt/wt)	Mean surface diameter		Moisture Content (%)*
		Wet *	Dry*	
NV	0:1	1.53 ± 0.02	0.0848 ± 0.0002	19.526 ± 0.01
NV1	0.5:1	2.74 ± 0.03	0.0105 ± 0.0002	12.806 ± 0.01
NV2	0.75:1	2.85 ± 0.05	0.0967 ± 0.0004	16.616 ± 0.02
NV3	1:1	3.04 ± 0.02	0.0907 ± 0.0003	17.883 ± 0.02

* All the values are expressed as mean ± SE

## *IN-VITRO* CHARACTERIZATION

### Buoyancy property

The time between the introduction of the beads into the medium and its buoyancy to the upper one-third of the dissolution vessel and the time for which the formulation constantly floated on the surface of the medium (duration of buoyancy) were measured simultaneously.[28]

### Percentage drug loading

An accurately weighed sample of beads (10 mg) was crushed in a mortar and added to 10 ml of 0.01N HCl. This mixture was centrifuged at 4200 rpm for 30 minutes, filtered and analyzed spectrophotometrically at λmax 281 nm against 0.01N HCl[28] as blank. The above-mentioned procedure was carried out with the blank beads. This was calculated by dividing the amount of drug in the sample beads by weight of the beads.

$$\%\;\mathrm{Drug}\;\mathrm{loading}\;=\;\left(\mathrm{Amount}\;\mathrm{od}\;\mathrm{drug}\;\mathrm{present}\right)/\left(\mathrm{Total}\;\mathrm{weight}\;\mathrm{of}\;\mathrm{beads}\right)\;\times \;100.$$

### Particle size

The particle size distribution of the beads was evaluated by sieve analysis.[29] One hundred grams of the beads were weighed and sieved through a set of sieves No: (12, 16, 18, 22, and 25) on a vibratory sieve shaker (PritecAC-99, M.B. Instruments, Delhi-7, India.) for 20 minutes, and the weight distribution was determined.

### Drug entrapment efficiency

An accurately weighed sample of beads (10 mg) was crushed in a mortar and added to 10 ml of 0.01N HCl. This mixture was sonicated for 30 minutes, filtered and analyzed spectrophotometrically at λmax 281nm against 0.01N HCl as blank.[30]

$$\%\mathrm{EE}\;=\;\left(\mathrm{Amount}\;\mathrm{of}\;\mathrm{added}\;\mathrm{drug}\;-\;\mathrm{Amount}\;\mathrm{of}\;\mathrm{non}\;\mathrm{encapsulated}\;\mathrm{drug}\right)/\left(\mathrm{Amount}\;\mathrm{of}\;\mathrm{added}\;\mathrm{drug}\right)\times 100.$$

### Moisture content

The moisture content of the formulated beads was determined using the procedure.[31] The moisture in a wet solid is that calculated on a dry weight basis; this value is referred to as moisture content.

$$\%\;\mathrm{Moisture}\;\mathrm{content}\;=\;\left(\mathrm{wt}.\;\mathrm{of}\;\mathrm{water}\;\mathrm{in}\;\mathrm{sample}\right)/\left(\mathrm{wt}.\;\mathrm{of}\;\mathrm{dry}\;\mathrm{sample}\right)\;\times \;100.$$

### Infrared (IR) spectroscopy analysis

The sample preparation includes grinding a quantity of the sample with a specially purified salt (usually potassium bromide) finely (to remove scattering effects from large crystals). This powder mixture is then crushed in a mechanical die press to form a translucent pellet,[32] through which the beam of the spectrometer can pass. This makes the observations of chemical reactions and processes quicker and more accurate.

### *In-vitro* release studies

An accurately weighed sample (40 mg) of a floating bead formulation was dropped in 900ml of 0.01 N HCl,[28] maintained at a temperature of 37°C ± 0.5°C and stirred at a speed of 50 rpm using USP dissolution apparatus type I (Basket). At different time intervals, a 10 ml aliquot of the sample was withdrawn and the same volume was replaced with an equal amount of plain dissolution medium. The collected samples were filtered and analyzed at λmax 281 nm, using a UV spectrophotometer against the medium buffer as a blank.

## RESULTS AND DISCUSSION

### Drug Entrapment efficiency (EE)

The percentage of drug entrapment efficiency was found to be (85.33, 75.66, 69.00, and 64.33) for the ratios of 0 : 1, 0.5 : 1, 0.75 : 1, and 1 : 1 by increasing the concentration of the CaCO_3_: alginate ratio. There was a decrease in drug entrapment because of the release of CO_2_. The reaction between CaCO_3_ and acetic acid led to the release of CO_2_, which penetrated the matrix of the alginate. Thus the porous beads resulted in decreased entrapment efficiency of the drug. The bead without CaCO_3_ attained high EE, which could be because of the highly dense internal structure of the alginate matrix.[28]

### Buoyancy properties

The floating ability was carried out for 24 hours and the data is tabulated in Table 2. We could see partial sinking in the formulation of NV1, but the formulations of NV2 and NV3 were floating completely throughout the study period. This might be due to the high concentration of the gas forming agent, because the floating ability was found to be directly related to the gas content of the matrix.[28]

**Table 2:** Evaluation parameters of various Nevirapine floating bead formulations (NV-No gas forming agent, NV1-0.5% gas forming agent, NV2-0.75% gas forming agent, NV3-1:1 gas forming agent) Completely sink (+ +), partially sink (+, completely float (- -)

Formulation	Floatation Property	Duration floatation	% drug loading*	% drug entrapment*	% release*
NV	(+ +)	24 hrs	35.87 ± 0.024	85.33 ± 3.29	17.03 ± 0.03
NV1	(+ -)	24 hrs	31.07 ± 0.028	75.66 ± 2.86	18.32 ± 0.03
NV2	(- -)	24 hrs	27.66 ± 0.020	69 ± 2.44	19.69 ± 0.02
NV3	(- -)	24 hrs	23.60 ± 0.036	64.33 ± 3.68	26.82 ± 0.02

* All the values are expressed as mean ± SE

### Particle size

**Figure 1:**
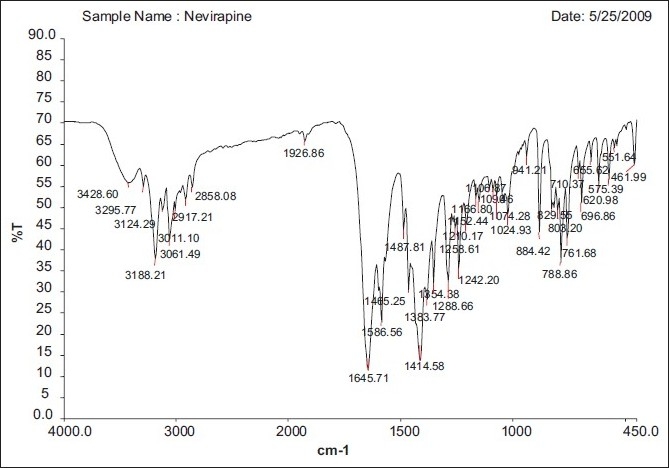
Nevirapine (pure)

The optical microscope images show that the formulated beads are spherical in shape and some show some rough surfaces [Figure 1], the particle size is determined by the sieve technique, using standard sieves (mesh) on an electrically equipped shaker for 15 minutes. The settled fractions were collected after weighing them individually, the particle size was calculated, and the mean particle size was between the range of 0.089 and 1.052 mm.

### Drug loading

The percentage drug loading of the different floating beads of nevirapine ranged between 23.6 and 35.87. It could be seen that by increasing the concentration of the gas forming agent, there was a decrease in drug loading.[28]

### Moisture content

The moisture content of the formulated beads was calculated by the proposed procedure.[31] The moisture in a wet solid was calculated on a dry weight basis, it was between 12.806 and 19.526 [Table 1].

### FT-IR spectral analysis

**Figure 2:**
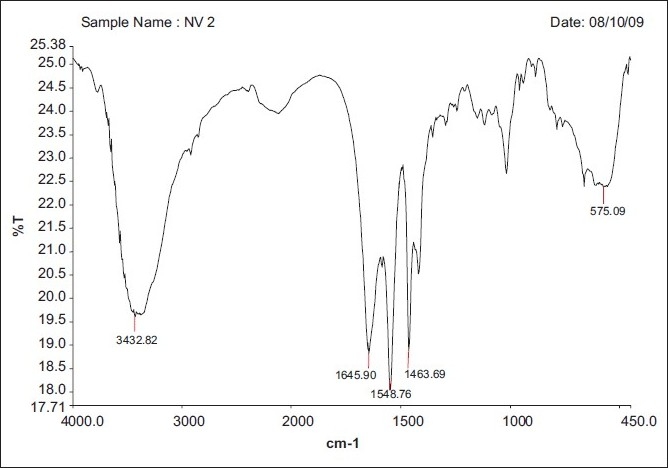
Blank floating Beads (without drugs)

**Figure 3:**
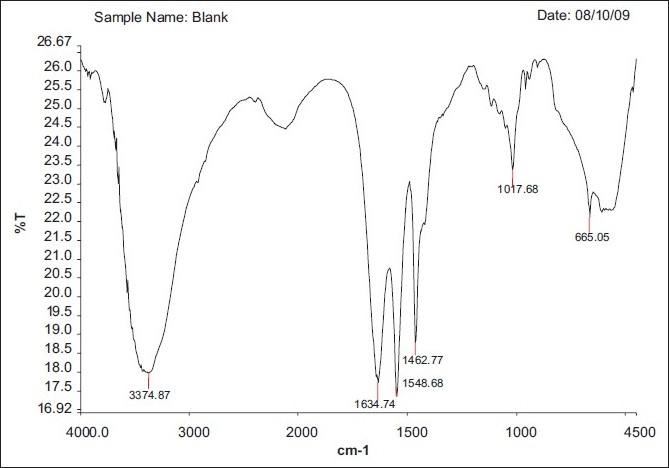
NV2 (0.75:1)^Table 1^

**Figure 4:**
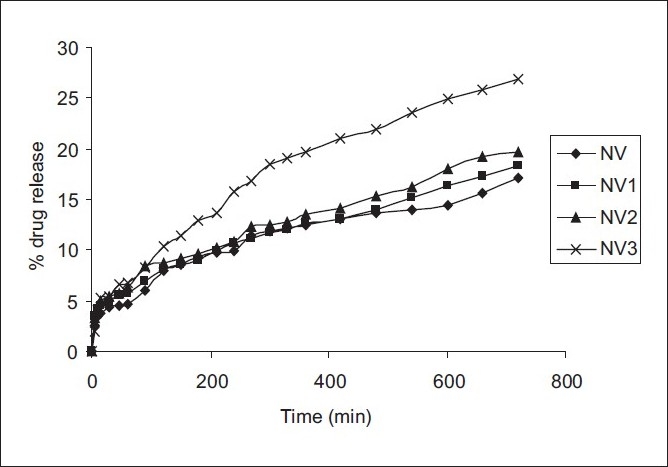
Effect of concentration of gas forming agent on *in-vitro* release of nevirapine from prepared floating beads

The FT-IR Spectrum of formulation NV2 is shown in Figures 2–4. The spectra reveal drug characteristics such as C=O (3432.82), N=C (1645.90) and NH (1548.76). The NH bond in plane (1463.69) shows the presence of the drug, with no interaction, and also the disappearance of some of the peaks in the formulation, which could be the encapsulation of the sodium alginate polymer.

### *In-vitro* drug release

**Figure 5:**
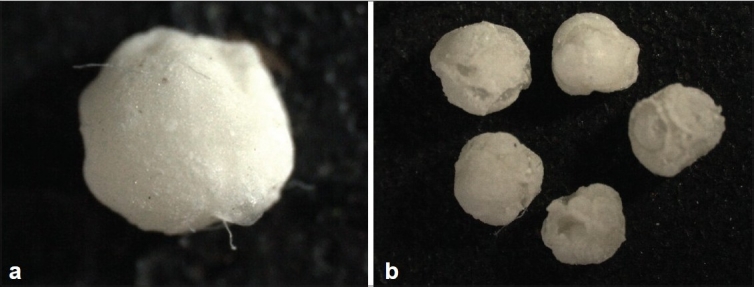
(a) Nevirapine NV3 (b) Floating beads NV2a

The nevirapine-loaded alginate beads were evaluated for drug release using 0.01NHCl (pH 1.2) as the dissolution media. The percentage drug release was (17.03, 18.32, 19.69, 26.82) for NV, NV1, NV2, and NV3, respectively, up to 12 hours [Figure 5], by using USP dissolution apparatus type-I (Basket). There was an increase in the release rate, with an increase of the gas-forming agent (CaCO_3_). This could be due to the pores present on the surface of the beads. Moreover, because of the more effective entrapment of the drug in the highly dense external structure of the alginate, the beads exhibited slow and extended release of the drug after 12 hours. The increased density of the polymer matrix at higher concentrations resulted in an increased diffusional path length, and due to this there could be a decrease in the overall drug release.

The gas forming agent was added at various concentrations to make the beads float in the medium. It was absorbed by the increased concentration of the gas forming agent, CaCO_3,_ and keeping the alginate at a constant, from 0.5 : 1 (NV1) to 1 : 1(NV3) there was an increase in buoyancy and there was an increase in drug release when compared with the formulation without the gas forming agent 0 : 1 (NV) (17.03%).

The data obtained for *in-vitro* release were fitted into equations for the zero order and first order, in the Higuchi, Korsmeyer, and Hixson release models; the interpretation of the data was based on the value of the resulting regression co-efficient [Table 3]. The *in-vitro* drug release showed the highest regression value for the Higuchi model, indicating diffraction to be the predominant mechanism of drug release.

**Table 3:** Release kinetics of drug release from Nevirapine floating beads (NV-No gas forming agent, NV1-0.5% gas forming agent, NV2-0.75% gas forming agent, NV3-1:1 gas forming agent)

Ratio	NV	NV1	NV2	NV3
Zero order	0.9219	0.9719	0.9726	0.9482
First order	0.8513	0.8513	0.8513	0.8513
Higuchi	0.9771	0.9721	0.9621	0.9953
Korsmeyer	0.9747	0.9635	0.9695	0.9789
Hixson	0.9284	0.9763	0.9773	0.9583

## CONCLUSION

It was observed that the formulation that contained the gas forming agent and drug ratio NV2 (0.75 : 1) was optimum, with respect to the floating ability, prolonged and sustained release, which was confirmed, as it obeyed the Higuchi (NV2) release kinetics. This above-mentioned formulation (NV2) could be a suitable composition for nevirapine, as a floating gastroretentive dosage form. To better understand the mechanism and drug release, *in-vivo* studies will have to be carried out in future.
